# Supplementary material for: The Association between Immune Checkpoint Proteins and Therapy Outcomes in Acute Myeloid Leukaemia Patients
Source: Cancers (Basel). 2023 Sep 9;15(18):4487. doi: 10.3390/cancers15184487 (PMC10526931; doi:10.3390/cancers15184487)
Supplement: Supplementary file 1 [file cancers-15-04487-s001.zip › cancers-2573996-supplementary.pdf]

Supplementary Table S1. Panel of antibodies used in flow cytometry.

Antibody and Fluorochrome	Producer	Clone	Volume per 200 µL
CD33 FITC	Becton Dickinson	P67.6	4 uL
CD3 PE-Cy7	BioLegend	UCHT1	2 uL
CD152 (CTLA-4) APC	Becton Dickinson	BNI3	5 uL
CD276 (B7-H3) PerCP- Cy5.5	BioLegend	MIH42	4 uL
CD274 (PD-L1) BV510	BioLegend	29E2A3	5 uL
CD279 (PD-1) PE	Becton Dickinson	EH12.1	0,5 uL

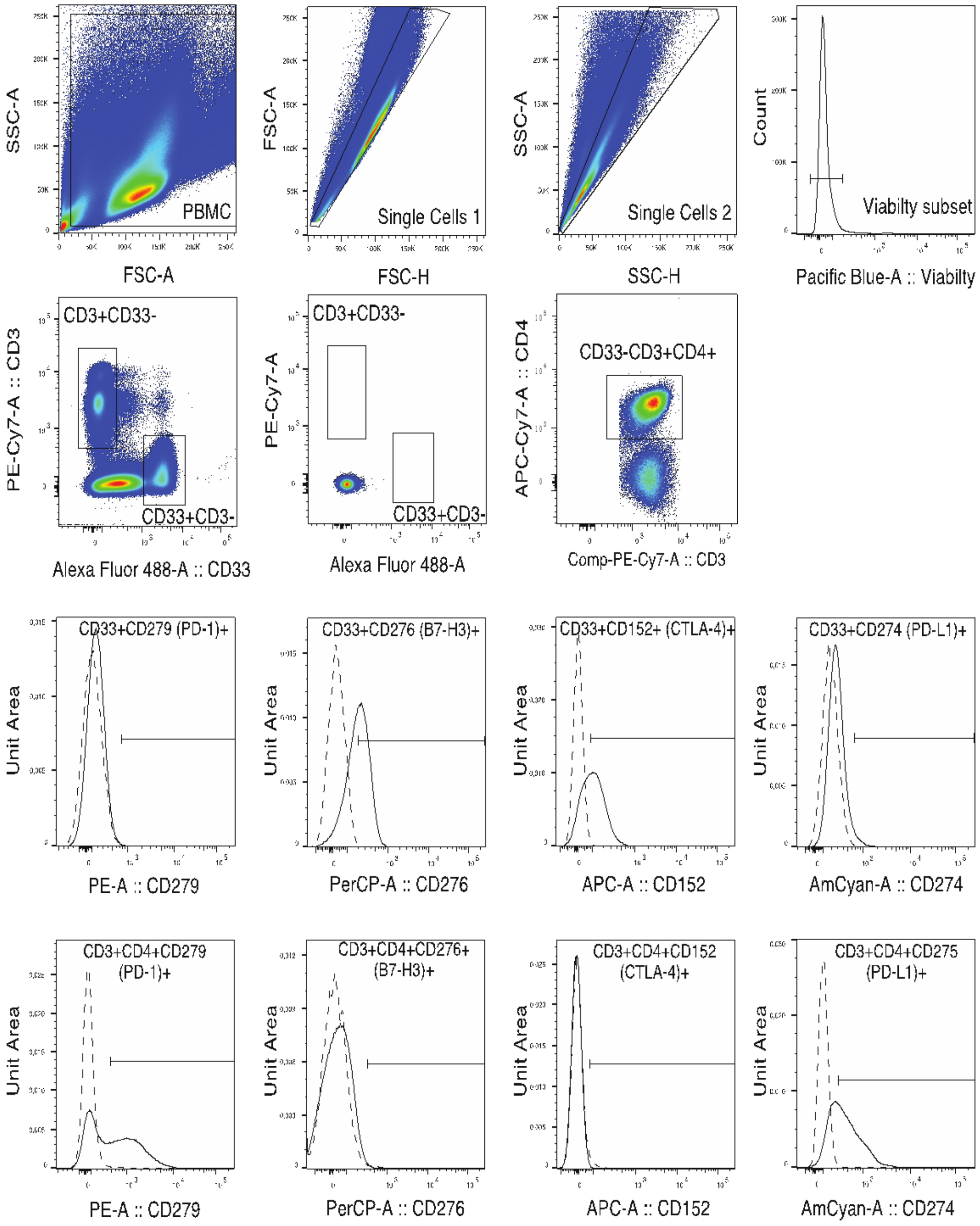

Supplementary Figure S1. Gating strategy implemented in the study panel.
